# Mild inhibition of mitochondrial complex I reduces Tau pathology and improves energy homeostasis and synaptic function in human‐derived neurons of LOAD patients

**DOI:** 10.1002/alz.084267

**Published:** 2025-01-03

**Authors:** Thi Kim Oanh Nguyen, Md Fayad Hasan, Eugenia Trushina

**Affiliations:** ^1^ Department of Neurology, Mayo Clinic, Rochester, MN USA; ^2^ Department of Molecular Pharmacology and Experimental Therapeutics, Mayo Clinic, Rochester, MN USA

## Abstract

**Background:**

Despite recent FDA approvement of disease‐modifying treatments that reduce Aβ, the identification of novel therapeutic strategies that could delay the Alzheimer’s disease (AD) development are needed. We identified and developed novel small molecule compounds that mildly inhibit mitochondrial complex I (MCI). Chronic treatment with a tool compound CP2 in 4 mouse models of familial AD was efficacious protecting against synaptic dysfunction and memory impairment, improving brain energetics and cognitive performance, reducing levels of human pTau and Ab. The objective of this study was to validate efficacy of this approach in a human brain‐like environment to support its translation into clinical trials. Human induced pluripotent stem cells (hiPSC) retain the host’s endogenous AD‐associated genetic alterations and recapitulate multiple hallmarks of late‐onset Alzheimer’s disease (LOAD).

**Method:**

Using pure human induced neurons (iNs) from *ApoE4/4*‐carrying LOAD patients, we evaluated mitochondrial bioenergetics and morphology using a Seahorse XFe96 flux analyzer and electron microscopy. Axonal trafficking was assessed using MitoTracker Red and live confocal imaging. Immunohistochemistry, quantitative‐PCR, and western blot were carried out to assess changes in the expression of genes and proteins, and cell viability assays were used to perform H_2_O_2_ challenge.

**Results:**

Cultures of hiPSC‐derived neurons were established using a lentiviral delivery for tetracycline‐inducible expression of NGN2‐GFP fusion gene driven by tetracycline‐responsive promoter. The validation of NGN2‐iNs confirmed the development of mature molecular, cellular, and synaptic properties. LOAD iNs have a significant reduction in the motile mitochondria in axons, increased accumulation of pTau, and altered bioenergetics compared to the control neurons. At CP2 concentrations relevant to *in vivo* treatment in APP/PS1 and 3xTgAD mice, significant reduction of pTau was associated with improved cellular bioenergetics, restored mitochondrial motility, and increased resistance to H_2_O_2_ induced oxidative stress.

**Conclusion:**

LOAD neurons carrying *ApoE4/4* alleles recapitulate LOAD phenotype allowing validation of a novel mitochondria‐targeted therapeutics. These data support previous observations in AD mice demonstrating that mild inhibition of MCI induce mild energetic stress with subsequent activation of multifaceted adaptive stress response and neuroprotective mechanisms improving cellular energetics and mitochondrial morphofunction in LOAD human neurons. Further efforts will be focused on the determination of mechanisms of neuroprotection.

